# Live cell imaging of plant infection provides new insight into the biology of pathogenesis by the rice blast fungus *Magnaporthe oryzae*

**DOI:** 10.1111/jmi.13382

**Published:** 2025-01-11

**Authors:** Berlaine G. Quime, Lauren S. Ryder, Nicholas J. Talbot

**Affiliations:** ^1^ The Sainsbury Laboratory University of East Anglia Norwich UK

**Keywords:** appressorium, biotrophy, effectors, pathogen, plant immunity, septin

## Abstract

*Magnaporthe oryzae* is the causal agent of rice blast, one of the most serious diseases affecting rice cultivation around the world. During plant infection, *M. oryzae* forms a specialised infection structure called an appressorium. The appressorium forms in response to the hydrophobic leaf surface and relies on multiple signalling pathways, including a MAP kinase phosphorelay and cAMP‐dependent signalling, integrated with cell cycle control and autophagic cell death of the conidium. Together, these pathways regulate appressorium morphogenesis.The appressorium generates enormous turgor, applied as mechanical force to breach the rice cuticle. Re‐polarisation of the appressorium requires a turgor‐dependent sensor kinase which senses when a critical threshold of turgor has been reached to initiate septin‐dependent re‐polarisation of the appressorium and plant infection. Invasive growth then requires differential expression and secretion of a large repertoire of effector proteins secreted by distinct secretory pathways depending on their destination, which is also governed by codon usage and tRNA thiolation. Cytoplasmic effectors require an unconventional Golgi‐independent secretory pathway and evidence suggests that clathrin‐mediated endocytosis is necessary for their delivery into plant cells. The blast fungus then develops a transpressorium, a specific invasion structure used to move from cell‐to‐cell using pit field sites containing plasmodesmata, to facilitate its spread in plant tissue. This is controlled by the same MAP kinase signalling pathway as appressorium development and requires septin‐dependent hyphal constriction. Recent progress in understanding the mechanisms of rice infection by this devastating pathogen using live cell imaging procedures are presented.

## LAY DESCRIPTION

Plant diseases claim about 30% of the global harvest of all crops. Controlling plant diseases in a sustainable manner is therefore vital to ensure global food security. Recent advances in live cell imaging have provided new insight into the biology of plant disease. This review describes progress made in understanding the cell biology of plant infection by the rice blast fungus *Magnaporthe oryzae*. Rice blast is a devastating disease but one that has emerged as a model system for understanding fungal infection of plants. Rapid advances have been made in our understanding of appressorium morphogenesis and plant tissue colonisation by the blast fungus. New methods to quantify membrane tension, turgor generation and to visualise cell‐to‐cell movement by the fungus are described. A model is presented that integrates the latest evidence of how fungal effectors are secreted and delivered into plant cells during disease development.

## 1 INTRODUCTION

Rice blast disease is one of the most important constraints on global rice production. The disease is caused by the filamentous hemibiotrophic fungus *Magnaporthe oryzae* (syn. *Pyricularia oryzae*), which has emerged as an important model organism for studying plant–fungal interactions.^1^ Losses to rice blast each year are estimated to be 6% of the global harvest^2^ but epidemics often cause up to 30% yield losses.^3^, ^4^ As more than half of the world's population rely on rice for their main calorific intake,^5^ blast disease represents a continual threat to global food security. In addition, *M. oryzae* can infect more than fifty grass species,^6^, ^7^, ^8^ leading to emergence of new diseases such as wheat blast, which emerged in Brazil after a host jump from a grass‐infecting isolate of *M. oryzae*.^9^ Having spread into neighbouring countries in South America, wheat blast appeared in Bangladesh in 2016^10^ where it now threatens the Indian subcontinent, a vital wheat‐growing region of the world,^11^ and more recently appeared in Zambia with the potential now to spread across Africa (Latorre et al., 2023).^12^ Globalisation, climate change and intense cultivation of cereal monocultures therefore make blast disease outbreaks more likely on wheat, rice, millets, oats and barley so the disease has the potential to become increasingly important to world agriculture.

In order to control blast disease, it is imperative that the biology of blast is better understood. Breakthroughs in live‐cell imaging, coupled with molecular genetic and genomic analysis of *M. oryzae*, have provided new insight into the cell biology of invasive growth by the fungus.^13^, ^14^, ^15^ In this review, we describe the major morphological transitions that the fungus undergoes during plant infection. We then critically evaluate studies of appressorium development, host cell penetration and intracellular colonisation by the blast fungus and review biological consequences of fungal infection induced during disease progression.

## 2 THE ADVENT OF LIVE‐CELL IMAGING OF PLANT INFECTION by *M. oryzae*

Historically, investigations of host plant infection by *M. oryzae* were made on fixed plant tissues^16^, ^17^, ^18^, ^19^, ^20^ and while these revealed the development of specialised invasive hyphae by the fungus,^16^ they did not allow the dynamics of the plant‐pathogen interaction to be captured. To observe the infection process of *M. oryzae* in living tissues, the leaf sheath inoculation method was developed^21^, ^22^ and is now widely used. This provides a simple and effective means of studying fungal growth in living plant tissue.^23^ Infecting leaf sheath tissues, which are devoid of chlorophyll, eliminates the need to clear tissues prior to imaging providing optically clear conditions to visualise each stage of fungal infection. Because *M. oryzae* is also easy to genetically transform, the visualisation of functional fluorescent fusion proteins is also widely used (for a video review of their use see Ref. [13], as well as cytological fluorescent dyes.^24^, ^25^ Development of these methods, coupled with use of confocal laser scanning microscopy, super‐resolution imaging, and electron microscopy, has enabled completely new insights into the major cellular changes that occur during blast infection.^23^, ^26^, ^27^, ^28^, ^29^, ^30^, ^31^ Live cell imaging has, indeed, proven revolutionary in our understanding of blast disease.

## 3 APPRESSORIUM FORMATION BY THE BLAST FUNGUS

**FIGURE 1:**
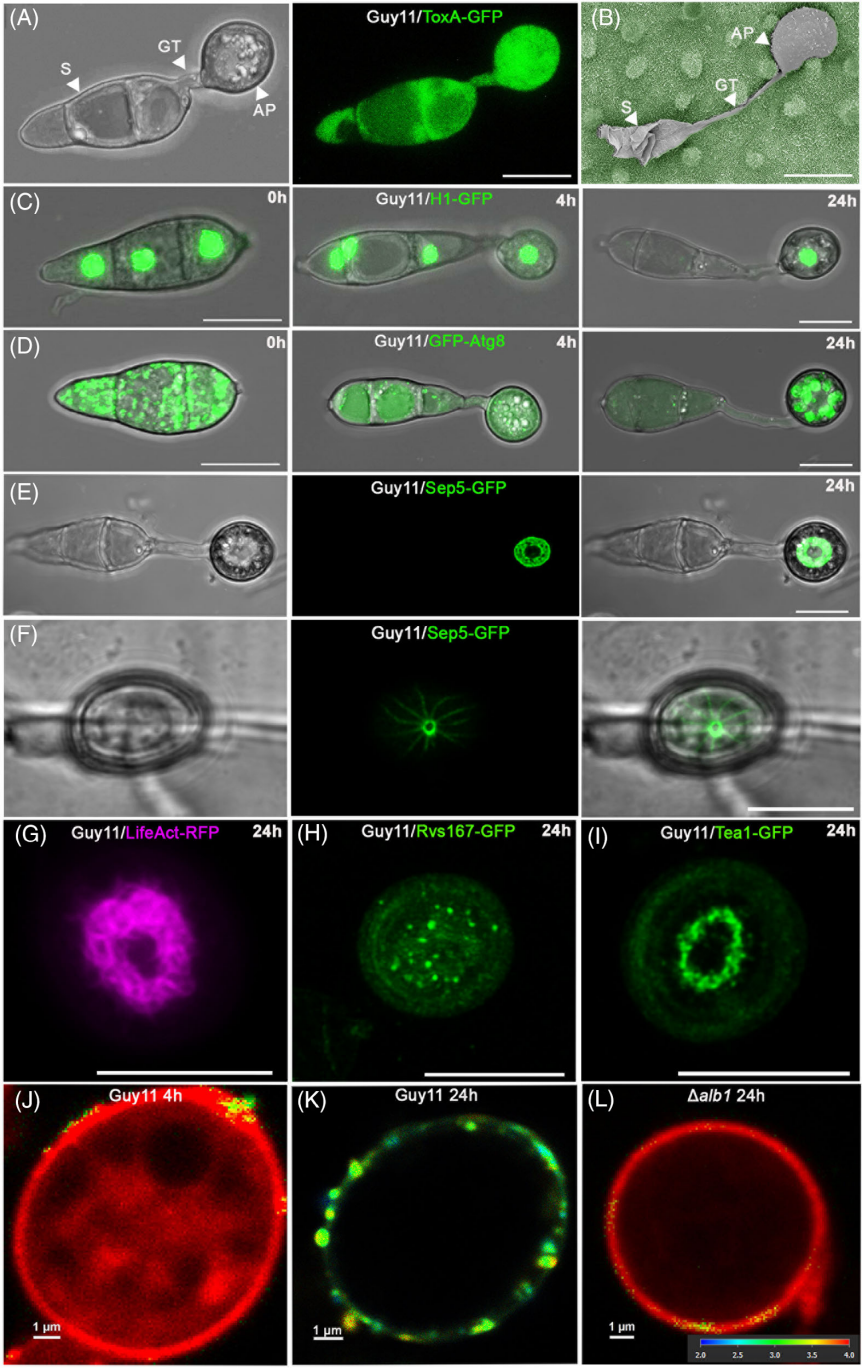
Infection‐related morphogenesis in the rice blast fungus *Magnaporthe oryzae*. (A) Confocal image of appressorium development by *M. oryzae* wild type strain Guy11 expressing cytoplasmic ToxA‐GFP. Conidia were germinated on glass coverslips and visualised 6 h post inoculation (hpi). The images represent maximum intensity projections of Z‐stack series captured on a Leica SP8 confocal laser scanning microscope. Scale bar = 10 µm. (B) Scanning electron micrograph with false colouring of a dome‐shaped appressorium (grey) on a rice leaf surface (green), freeze dried 24 h after inoculation. The contents of the spore undergo autophagy and are recycled to the incipient appressorium, resulting in enormous turgor that is translated into mechanical force to penetrate the waxy rice leaf cuticle. Scale bar = 10 µm. (C) Conidia were harvested from Guy11 expressing H1‐GFP and inoculated onto glass coverslips. Images are maximum projections of Z‐stack series captured on a Leica SP8 confocal laser scanning microscope at 0, 4 and 24 hpi. Scale bar = 10 µm. (D) Conidia were harvested from Guy11 expressing GFP‐Atg8 and inoculated onto glass coverslips. Images are maximum projections of Z‐stack series captured on a Leica SP8 confocal laser scanning microscope at 0, 4 and 24 hpi. Scale bar = 10 µm. (E) Organisation of Sep5‐GFP in the appressorium pore of Guy11 at 24 hpi on glass coverslips. Scale bar = 10 µm. (F) Organisation of Sep5‐GFP in the appressorium pore of Guy11 at 24 hpi on rice leaf sheath cultivar Moukoto. Scale bar = 10 µm. (G) Organisation of actin with LifeAct‐RFP in the appressorium pore of Guy11 at 24 hpi. (H) Organisation of Bin‐Amphiphysin‐Rvs (BAR) domain protein Rvs167‐GFP in the appressorium pore of Guy11 at 24 hpi. (I) Organisation actin‐binding protein Tea1‐GFP in the appressorium pore at 24 hpi. Scale bars = 10 µm. (J) FLIM image of Guy11 appressorium at 4 hpi stained with N^+^‐BDP rotor probe. Using FLIM, this rotor probe is able to detect differences in plasma membrane tension of appressoria during infection‐related‐development in wild type strain Guy11 and melanin deficient mutant *alb1^−^*. Red = low tension, green = high tension. (K) FLIM image of Guy11 appressorium at 24 hpi stained with N^+^‐BDP rotor probe. (L) FLIM image of melanin‐deficient mutant *alb1^−^* appressorium at 24 hpi stained with N^+^‐BDP rotor probe. The colour corresponds to fluorescence lifetime values expressed in nanoseconds, as shown in the key 2–4 ns. Scale bar = 1 µm. S = spore, GT = germ tube and AP = appressorium. Conidial germination onto glass coverslips (A, C, D, E, G, H, I) and leaf sheath infection (F) were incubated at 26°C and 24°C, respectively.

Plant infection by the blast fungus proceeds when a three‐celled conidium lands and attaches to the hydrophobic leaf surface via spore tip mucilage released from its apex.^32^ The spore adheres tightly from its tip and germinates rapidly on the leaf surface to form a polarised germ tube within 2 h. The germ tube then hooks and flattens at its tip before differentiating into an appressorium, a dome‐shaped infection structure (see Figure 1A and B) with a specialised cell wall containing a layer rich in chitin and a thick layer of melanin between the cell membrane and the cell wall.^32^, ^33^, ^34^, ^35^, ^36^ Appressorium development requires perception of an appropriate surface, which must be hard and hydrophobic, as well as free of exogenous nutrients. These conditions are perceived by surface receptors, such as the Pth11 G‐protein coupled receptor, the Msb2 and Sho1 proteins which act upstream of the Pmk1 mitogen‐activated protein kinase (MAPK) pathway, and the cyclic AMP‐dependent protein kinase A pathway, which are necessary for appressorium formation and function.^13^ Phosphorylation of the Pmk1 MAPK occurs within 1 h of spore germination and leads to phosphorylation of a large‐set of substrates including a novel regulator Vts1, and the Hox7, Far1 and Fkh1 transcription factors.^15^ Phosphorylation of these substrates ultimately results in major changes in gene expression in which 49% of the genes of *M. oryzae* are differentially regulated during appressorium morphogenesis.^37^ The appressorium undergoes intense melanin biosynthesis and turgor generation which requires high concentrations of intracellular compatible solutes such as glycerol.^38^ *M. oryzae* appressoria generate up to 8.0 MPa of pressure, which is applied at the leaf surface to enable a rigid penetration peg to breach the rice leaf cuticle and epidermal cell wall.^39^ Appressorium morphogenesis is also regulated by a series of cell cycle controls in which initiation of appressorium formation requires an S‐phase checkpoint, appressorium maturation requires mitotic entry, and completion of mitosis is necessary for development of a functionally competent appressorium.^40^ A metabolically regulated cell‐cycle checkpoint has also been reported to be necessary for appressorium morphogenesis involving inactivation of the target‐of‐rapamycin (TOR) kinase, which is maintained by the Asd4 GATA transcription factor, thereby repressing expression of genes involved in nitrogen assimilation to maintain low glutamine levels.^41^ Following mitosis, one daughter nucleus moves from the germ tube to the appressorium and the second daughter returns to the apical conidial cell (Figure 1C). The conidium then undergoes an autophagy‐dependent process leading to cell death.^42^, ^43^ This requires cargo‐independent autophagy and it has been demonstrated that proteins encoded by genes associated with nonselective autophagy are all required for pathogenicity.^42^, ^43^, ^44^ Autophagy is necessary for collapse of the three‐celled conidium and trafficking of its contents into the developing appressorium. Regulated cell death of the spore has, however, also been proposed to require ferroptosis^25^, ^45^ which results in lipid peroxidation and loss of viability of each cell of the conidium (see Figure 1D). This suggests that regulated cell death of the conidium requires autophagy but may not be caused by it directly, instead requiring another form of regulated cell death, although further study is essential to confirm these observations.

Appressoria initially undergo radial or isodiametric growth, expanding uniformly in all directions to form dome‐shaped infection structures for host penetration. Re‐polarisation of the appressorium then occurs at its base, where a specific region, the appressorium pore, is defined by a toroidal network of septin GTPases which generates cortical rigidity and re‐organises F‐actin at the point of plant infection.^46^ The septin ring forms during appressorium maturation marking the precise point of penetration peg emergence, as shown in Figure 1E. On a rice leaf surface, the septin ring forms in the same manner, but then undergoes further constriction to a diameter of approximately 0.9–1.1µm once the penetration peg is formed (Figure 1F). Septins are required for scaffolding F‐actin at the base of the appressorium^47^ as shown in Figure 1G. The septin ring also acts as a lateral diffusion barrier for polarity factors such as Bin‐Amphiphysin‐Rvs (BAR)‐domain proteins like Rvs167 (Figure 1H), the exocyst complex, and actin‐binding proteins such as Tea1 (Figure 1I).^46^, ^48^ In this way, rapid actin polymerisation, polarised exocytosis and cell wall biogenesis are focused to facilitate peg development and protrusive force generation at the base of the appressorium.

Recently, new tools have been developed to investigate appressorium turgor generation. Changes in membrane tension can be quantified via fluorescence lifetime imaging (FLIM) using a mechanosensor plasma membrane rotor probe, N^+^‐BDP, which can detect spatial variations in membrane tension in *M. oryzae* appressoria.^14^ An incipient appressorium at 4 h shows low and uniform membrane tension causing mechanical restriction of the rotor probe upon photoexcitation and longer average fluorescence lifetimes (Figure 1J), whereas by 24 h, an appressorium with high turgor exhibits high membrane tension, with a disordered membrane, allowing free rotation of the probe, resulting in shorter average fluorescent lifetimes, as shown in Figure 1K. Strikingly, the appressorium membrane under high tension also shows considerable heterogeneity, suggesting that there are regions varying considerably in membrane fluidity in the pressurised cell. By contrast, a nonpathogenic melanin‐deficient mutant *alb1* ^−^ was found to exhibit low spatially homogeneous tension (Figure 1L).^14^

## 4 INVASIVE GROWTH BY *M. oryzae* IN LIVING PLANT TISSUE

**FIGURE 2:**
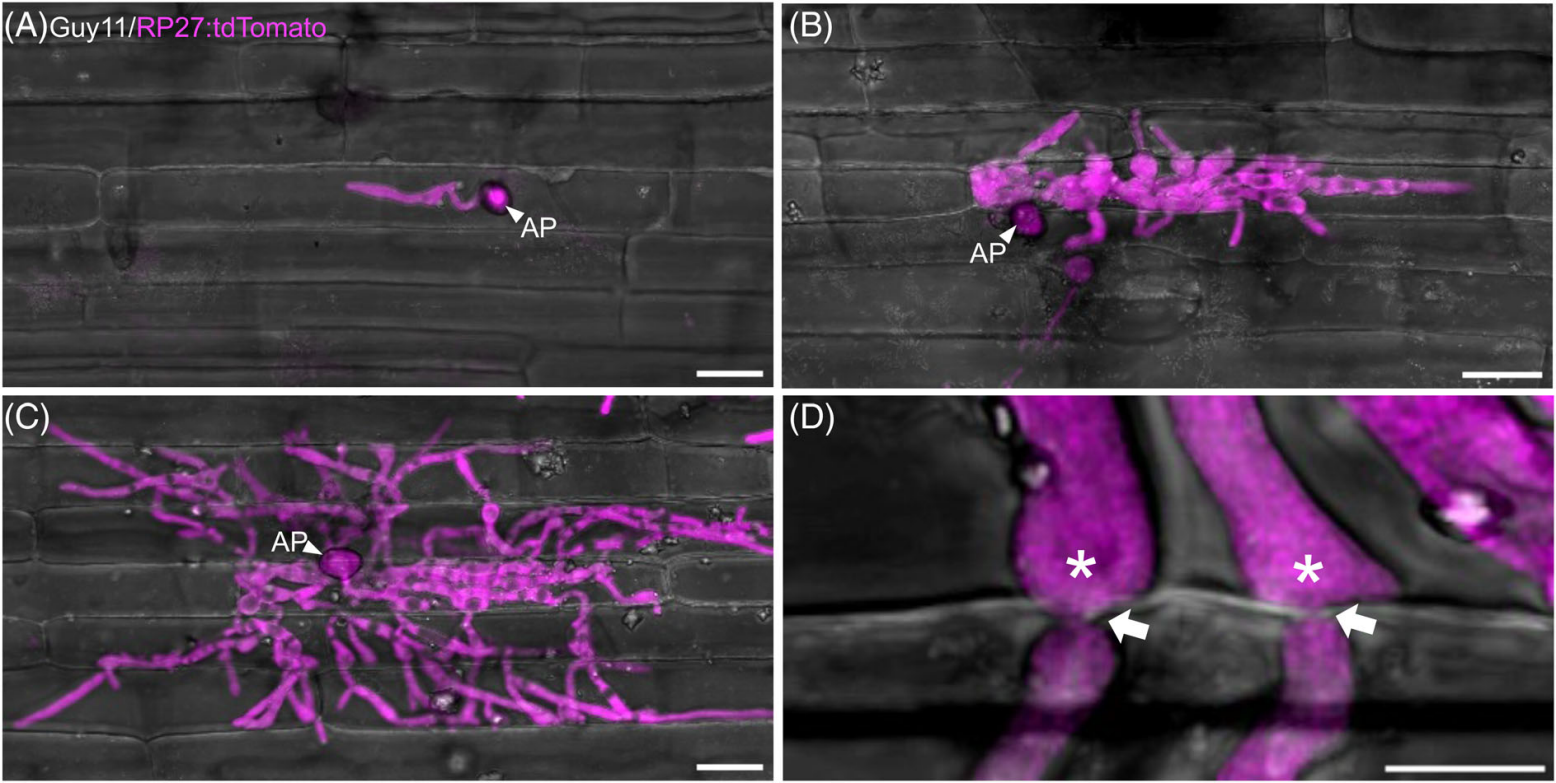
Progression of rice tissue invasion by *Magnaporthe oryzae*. Confocal images of of rice cultivar Kitaake leaf sheaths inoculated with *M. oryzae* Guy11 expressing tdTomato driven by *M. oryzae* ribosomal protein 27 (RP27), a constitutive promoter at (A) 24 h post‐inoculation (hpi), (B) 36 hpi and (C) 48 hpi. Scale bars = 20 µm. (D) Confocal image showing transpessoria (in asterisks), which enables *M. oryzae* to move from cell‐to‐cell. White arrows indicate crossing points and hyphal constrictions as the fungus moves to the neighbouring host cell. Scale bar = 5 µm. Leaf sheath infections for all time points were incubated at 24°C. All images shown are maximum projections of Z‐stack series taken using Leica SP8 confocal laser scanning microscope. AP = appressorium.

Once the fungus has punctured the leaf surface, it extends a narrow primary hypha that rapidly differentiates into thicker, bulbous invasive hyphae (IH) which colonise primary‐invaded cells before infecting neighbouring cells.^16^ Rapid plant tissue colonisation can be visualised from 24 h after inoculation using a *M. oryzae* strain expressing cytoplasmic red fluorescent protein (expressed using the high level constitutive promoter *M. oryzae* ribosomal protein 27 (RP27)^27^ driving tdTomato) as shown in Figure 2A. Invasive hyphae initially grow within the first invaded host cells, filling them as they grow and undergoing cortical scanning in which hyphal tips make contact with the edge of the plant cell to locate potential crossing points. To move to uninfected adjacent cells, invasive hyphae swell at the tip and then undergo severe hyphal constriction to a diameter of approximately 0.6‐0.8 µm (Cruz‐Mireles et al., 2021).^50^ Cell‐to‐cell crossing points located by invasive hyphae correspond to pit field sites, containing plasmodesmata^23^ and the fungus utilises these cell junctions to facilitate its spread. The fungus starts to move to neighbouring cells by 36 h after inoculation (Figure 2B) and colonises more cells by 48 h (Figure 2C). The process of cell‐to‐cell invasion is controlled by the Pmk1 MAPK, mirroring the process of appressorium development.^49^ When a conditional *pmk1* analogue‐sensitive mutant was generated, it was shown that inhibition of MAPK activity with the ATP analogue kinase inhibitor 1NA‐PP1 led to the fungus becoming trapped in the initial infected cell, unable to invade adjacent tissue. Furthermore, Pmk1 appears to regulate a large set of fungal proteins associated with septin and cytoskeletal re‐modelling and hyphal constriction, as well as effector‐encoding genes required for proliferation of the fungus in plant tissue.^50^ Cell‐to‐cell movement thus requires a specialised infection structure similar to the appressorium, that has been termed a transpressorium (Figure 2C). In this way, the fungus can cross pit field sites, allowing integrity of the plant cell membrane to be maintained in adjacent plant cells as they are invaded.^13^, ^50^, ^51^

## 5 RICE PLASMA MEMBRANE DYNAMICS DURING *M. oryzae* INVASION

**FIGURE 3:**
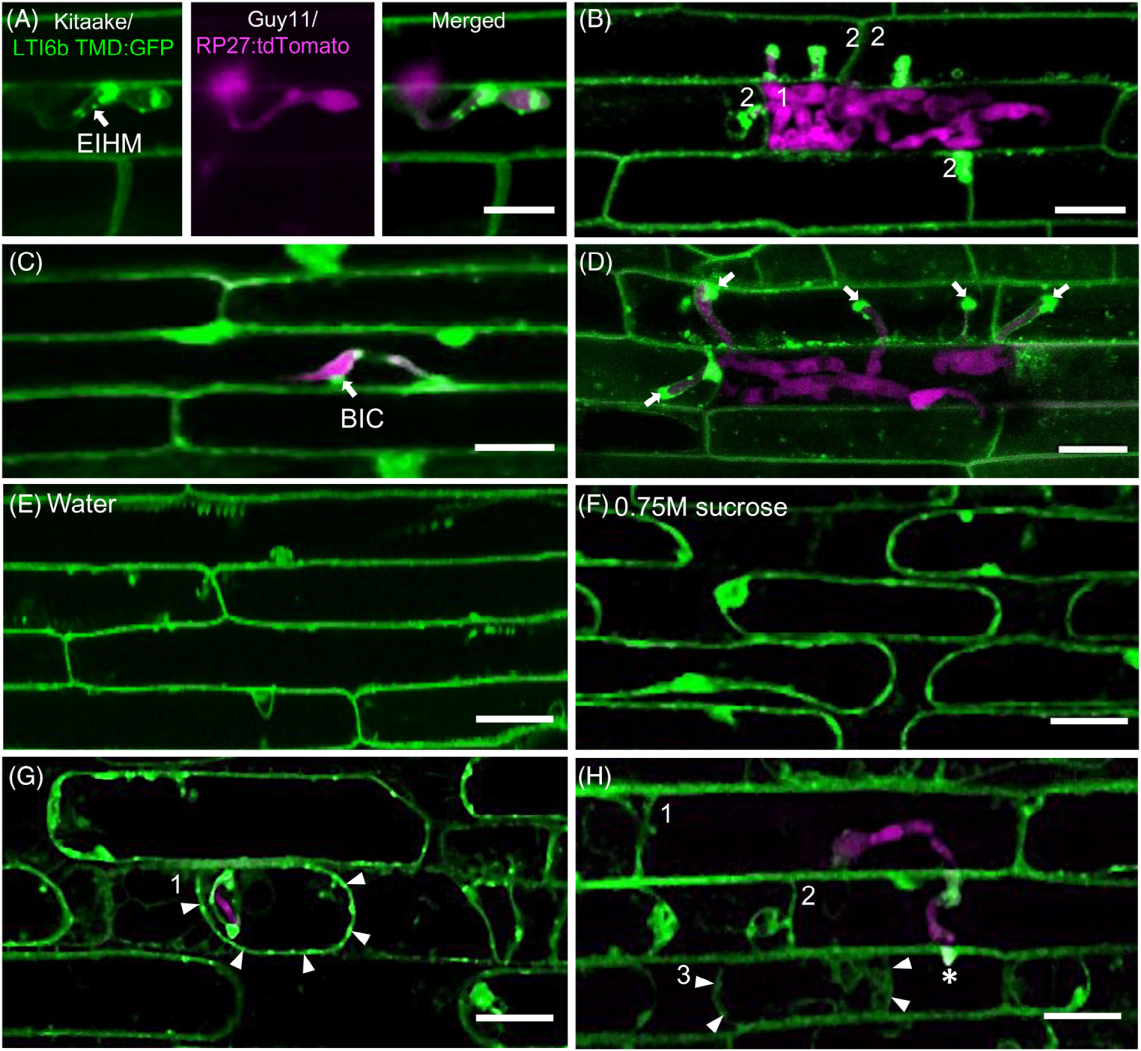
Host plasma membrane changes during *Magnaporthe oryzae* infection. (A‐B) Extra‐invasive hyphal membrane (EIHM) dynamics during *M. oryzae* infection. (A) Invasive hypha (IH) is enclosed with intact EIHM at 24 h post‐inoculation (hpi). (B) EIHM becomes disrupted in the initially invaded cell at 36 hpi. Numbers indicate the order by which the fungus invades rice cells. Confocal images were prepared from leaf sheath inoculations using rice transgenic line expressing the plasma membrane marker LTI6b TMD:GFP (green) and rice blast isolate Guy11 RP27:tdTomato (magenta). Scale bars = 20 µm. (C, D) Biotrophic interfacial complex (BIC) formation during *M. oryzae* infection. (C) BIC (indicated by white arrow) is formed as the fungus enters the first cell at 24 hpi. (D) BICs are formed in each invasive hypha as the fungus invades the neighbouring cells at 36 hpi. Confocal images were prepared from leaf sheath inoculations using rice transgenic line expressing the plasma membrane marker LTI6b TMD:GFP (green) and blast isolate Guy11 RP27:tdTomato (magenta). Scale bars = 20 µm. (E–H) Rice plasma membrane dynamics during *M. oryzae* infection. (E) Uninfected rice transgenic line expressing the plasma membrane marker LTI6b TMD:GFP mounted in water. (F) Plasmolysed, uninfected cells of the rice transgenic line LTI6b TMD:GFP mounted in 0.75M sucrose. (G) Plasmolysed initially infected cell indicates intactness of the rice plasma membrane at 24 hpi. (H) Previously invaded cells lose the ability to plasmolyse after the fungus has moved to the neighbouring cells due to loss of plasma membrane integrity. Numbers indicate the order by which the fungus invades the host cells. Confocal images were prepared from leaf sheath inoculations using rice transgenic line expressing the plasma membrane marker LTI6b TMD:GFP (green) and rice blast isolate Guy11 RP27:tdTomato (magenta). White arrowheads indicate the shifting of rice membranes away from the cell wall during plasmolysis. White asterisk indicates new penetration site. Scale bars = 20 µm. (A–H) Leaf sheath infections for all time points were incubated at 24°C. All images shown are maximum projections of Z‐stack series taken using Leica SP8 confocal laser scanning microscope.

Expression of fluorescent gene fusions in both rice and *M. oryzae* has recently allowed for much better definition of the fungal‐plant interface. Spatiotemporal changes in the rice plasma membrane (PM), for example, can now be directly visualised during *M. oryzae* infection. As the fungus enters the first invaded cell, it is enveloped by the rice plasma membrane, which forms a specialised compartment called the extra‐invasive hyphal membrane (EIHM) (Figure 3A). The EIHM surrounds invasive hyphae as they grow in the plant cell but as the fungus moves to previously unoccupied neighbouring cells, integrity of the EIHM in the first invaded cell is lost (Figure 3B). It has been demonstrated that the EIHM forms a sealed compartment that creates a barrier between the fungus and the host cytoplasm. Labelling with the lipophilic styryl dye FM4‐64 allows visualisation of the EIHM, and the inability of the dye to penetrate and label the invasive hyphae has provided evidence that the EIHM forms a sealed compartment.^23^ A membrane‐rich structure is also found at the EIHM called the biotrophic interfacial complex (BIC). This is a host‐derived structure that forms a distinct punctum where fungal effectors are concentrated. In *M. oryzae* strains expressing fluorescently‐tagged effector proteins, BIC structures can be visualised clearly. The BIC undergoes a two‐stage development as the fungus invades host cells during biotrophic growth. In newly invaded cells, the BIC appears as an EIHM membranous cap, termed a ‘tip BIC’ that can be observed at the primary hyphal tip.^23^ As the filamentous primary penetration hypha differentiates into a bulbous invasive hypha, the BIC develops into a distinctive structure beside the first invasive hyphal cell, where it has been termed a ‘side‐BIC’.^27^ The BIC forms when the fungus penetrates the initial host cell and is consistently visible 24 h after inoculation. The BIC can be readily visualised in rice transgenic lines expressing the plasma membrane marker LTI6b TMD:GFP, as a bright punctate structure as shown in Figure 3C and BICs can be observed as each invasive hypha moves into the next rice cell (Figure 3D). Strikingly, only a single BIC is produced in the initially invaded cell. However, as the fungus spreads to neighbouring cells by 36 h post‐inoculation, each invasive hypha that branches from the initial infection site forms a BIC in each newly colonised host cell. The host plasma membrane remains intact during fungal invasion of the initial cell but becomes disrupted as the fungus moves into the neighbouring cell. This can be observed using a plasmolysis assay, where the plasma membrane separates from the cell wall, making it more clearly visible.^23^, ^52^ To illustrate the loss of plasma membrane integrity, a plasmolysis assay was carried out on infected rice cells expressing the plasma membrane marker LTI6b‐GFP^28^ and used to evaluate plasma membrane integrity (Figure 3E). Using a rice transgenic line expressing the LTI6b TMD:GFP, healthy, uninfected rice cells mounted in 0.75M sucrose result in plasmolysed cells where the plasma membrane recedes from the cell wall due to hyperosmotic conditions (Figure 3F). In infected cells, initially invaded cells at 24 hpi also retain their ability to plasmolyse, consistent with host cell membrane integrity being maintained in fungal colonised cells. The EIHM was found also found to shrink around the invasive hypha (Figure 3G). However, as the fungus moves to neighbouring cells, the initial and subsequently invaded cells lose their ability to plasmolyse, indicating that the host plasma membrane is disrupted at this time. Only newly invaded rice cells can plasmolyse (Figure 3H), confirming that *M. oryzae* always colonises living plant cells as it spreads in rice tissue. Biotrophic growth therefore involves a mosaic pattern in which newly invaded plant tissue is alive with intact plasma membranes and a discrete EIHM bounding invasive hyphae, whereas cells from which the fungus spreads lose their viability as soon as adjacent cells are invaded, leading to host cell death. Hemibiotrophy in the blast fungus therefore does not involve distinct switches in growth habit at a precise point following infection, but rather is a consequence of the way in which invasive hyphae move, via transpressoria and pit fields, between host cells always with a growing zone of biotrophic development. As the infection progresses, *Magnaporthe* therefore predominantly appears to switch from a biotrophic phase in which host cells are alive, to a later necrotrophic phase where most cells that the fungus occupies are killed and their nutrients consumed. This leads to the characteristic lesions, sporulation and tissue death associated with rice blast disease.

## 6 SECRETION AND DEPLOYMENT OF *M. oryzae* EFFECTORS

**FIGURE 4:**
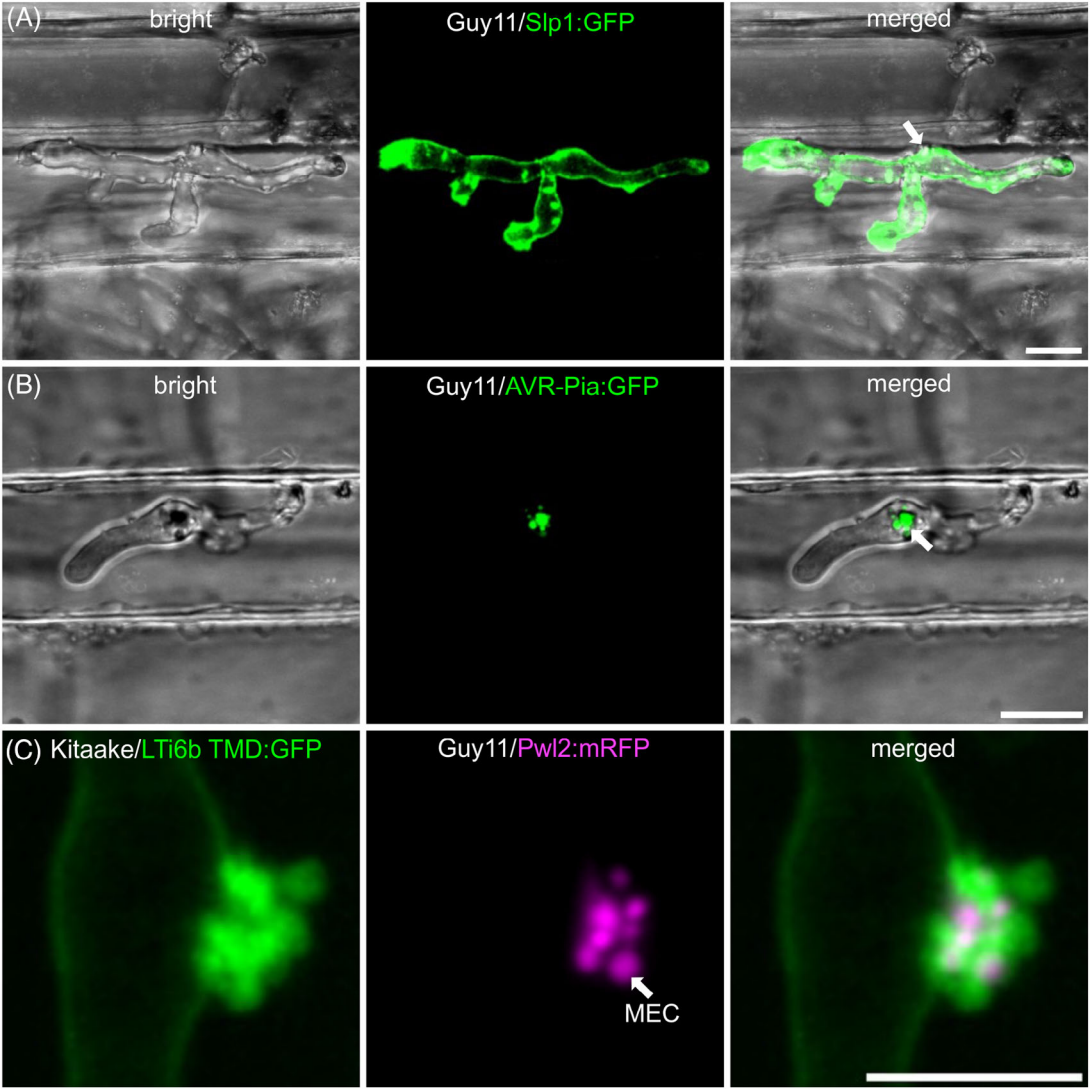
Localization of *M. oryzae* effectors during infection. (A) Confocal image shows Guy11 expressing Slp1:GFP, an apoplastic effector, which localizes at the apoplastic space. Note that there is no fluorescence signal in the BIC (white arrow) Scale bars = 10 µm. (B) Confocal image shows Guy11 expressing a known cytoplasmic effector AVR‐Pia:GFP. AVR‐Pia:GFP preferentially accumulated in the BIC (indicated by white arrow). (C) Confocal image shows a close‐up of the biotrophic interfacial complex (BIC). The BIC is plant plasma membrane‐derived and colocalizes with Guy11 expressing Pwl2:mRFP. Pwl2 is a known *M. oryzae* cytoplasmic effector localizing at the BIC. Scale bar = 5 µm. Leaf sheath infections were incubated at 24°C. All images were taken 26 h post‐inoculation (hpi) and are presented as maximum intensity projections of Z‐stack series captured using Leica SP8 confocal laser scanning microscope.

**FIGURE 5:**
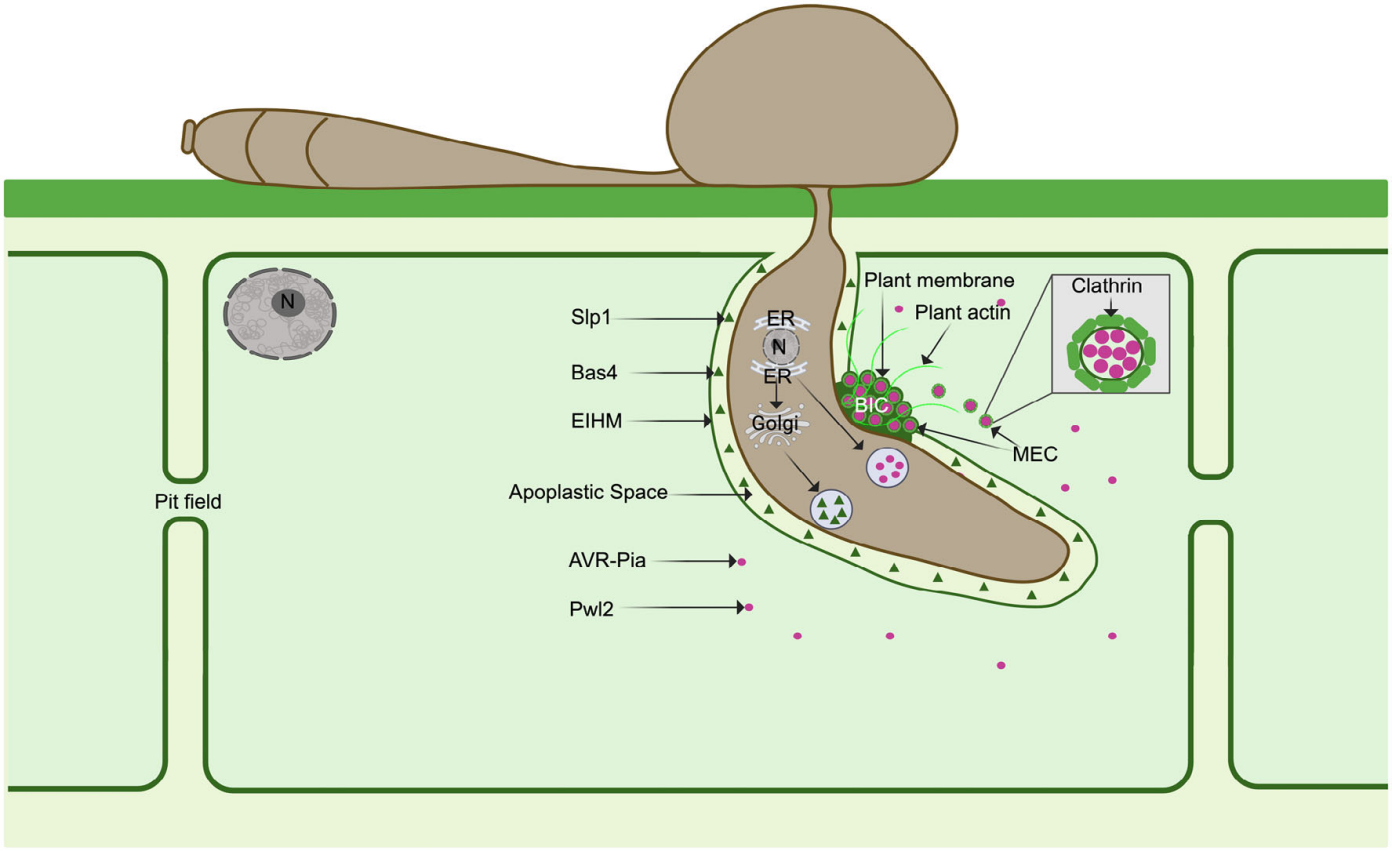
Model for the translocation of *M. oryzae* effectors into rice cells. During biotrophic growth, *M. oryzae* deploys a battery of effector proteins which are either apoplastic or cytoplasmic, to suppress host immune responses and facilitate fungal colonisation. Apoplastic effectors are secreted via the conventional ER‐to‐Golgi, Brefeldin A (BFA)‐sensitive pathway, and reside in the apoplastic or EIHM matrix (EIHMx) enclosed by the extra‐invasive hyphal membrane (EIHM). Cytoplasmic effectors are secreted using a nonconventional BFA‐insensitive pathway and accumulate in the biotrophic interfacial complex (BIC). Cytoplasmic effectors tagged with fluorescent proteins are observed to be packaged in membranous effector compartments (MECs) which co‐localise at the BIC with fluorescently‐tagged rice plasma membrane (LTI6b:GFP). Clathrin‐mediated endocytosis then results in MECs being taken into the cytoplasm of the host from where effectors are released to fulfil diverse immuno‐suppressive functions during fungal infection.

To successfully invade living host tissue, *M. oryzae* delivers a complex repertoire of effector proteins to manipulate plant immunity and protect the pathogen from defence responses.^30^, ^53^, ^54^, ^55^ A recent study revealed that 546 putative Magnaporthe effector‐encoding (*MEP*) genes are expressed during plant infection and show specific patterns of temporal co‐regulation.^31^ Structurally related but sequence‐unrelated effectors, such as the MAX (Magnaporthe Avrs and ToxB‐like) effectors and putative ADP‐ribosylation factor‐like effectors are expressed specifically between 24 and 48 h after infection. This suggests that effectors are under tight transcriptional control during infection. Consistent with this, a forward genetic screen to search for effector regulators identified *RGS1*, a previously described regulator of G‐protein signalling during appressorium development, as a transcriptional regulator of effector gene expression. Rgs1 represses effector expression during the prepenetration phase of development, enabling their specific de‐repression upon plant infection.^56^ Similarly, the Pmk1 MAP kinase that regulates transpressorium development is also necessary for expression of at least 50 effector‐encoding genes, including *SLP1*, *BAS1* and *BAS3*.^31^, ^49^ Fluorescent protein tagging has enabled categorisation of effectors based on their localisation patterns *in planta*. Apoplastic effectors delivered to the apoplast, such as *SLP1*,a LysM domain protein binding fungal cell wall chitin, appears around the periphery of invasive hyphae (Figure 4A), whereas cytoplasmic effectors, such as *AVR‐Pia*, are concentrated in the BIC before being translocated to the rice cytoplasm (Figure 4B). The reproducible visualisation of effector expression in the BIC,^27^, ^31^, ^57^ is consistent with a role for the BIC as an active site of effector translocation to the host cytoplasm. Evidence to support this hypothesis has been reported in a study which showed that cytoplasmic effectors are secreted in a manner that is insensitive to brefeldin A (BFA), suggesting an unconventional Golgi‐independent secretion process. By contrast, apoplastic effectors such as Slp1 and Bas4 are secreted in a BFA‐sensitive way, suggesting conventional secretion.^26^ Cytoplasmic effectors therefore appear to be secreted from the BIC‐associated cell, which is a modified hyphal tip in an exocyst‐dependent but Golgi‐independent manner. Thereafter they accumulate within the BIC outside of the fungal cell wall but still within the EIHM (Figure 4C). Unconventional secretion of *M*. oryzae furthermore requires tRNA thiolation and alternate codon usage of genes encoding effectors destined for delivery to the BIC.^58^ Following secretion, effectors can be visualised within the BIC. between the fungal cell wall and the EIHM, using super resolution imaging within punctate structures called membranous effector compartments (MECs), which have an initial diameter of up to 249 nm, but can enlarge/fuse to form larger 500–1000 nm MECs in mature BICs.^30^ MECs co‐localise with plant plasma membrane markers, such as LTI6b‐GFP and also with Clathrin light chain, suggesting a role for clathrin‐mediated endocytosis in effector uptake into plant cells from the BIC (Figure 5). Inhibiting clathrin‐mediated endocytosis using chemical inhibitors or by virus‐mediated gene silencing of OsCHC1 (Clathrin Heavy Chain 1) or OsAP2 (AP2/ERF transcription factor family) prevented effector uptake, whereas inhibition of clathrin independent endocytosis by silencing OsFLOT1 (Flotillin 1), or with chemical inhibitors, had no effect on effector uptake.^30^ Taken together, these studies suggest that effector secretion occurs via two routes in *M. oryzae*, depending on the destination of the effector. Cytoplasmic effectors are secreted unconventionally, accumulating in the BIC from where they are taken into plant cells through a mechanism involving clathrin‐mediated endocytosis. Interestingly, very similar results have been found in the oomycete pathogen *Phytophthora infestans*, which is only very distantly related to fungal pathogens such as *M. oryzae*. Cytoplasmic effectors of *P. infestans* are secreted in a BFA‐insensitive manner^59^ and clathrin‐mediated endocytosis is also implicated in their uptake during plant infection from haustoria even though a BIC structure is not observed. In *P. infestans* infections of the model host *Nicotiana benthamiana*, transient silencing of *NbCHC*, encoding clathrin heavy chain, or *NbAra6* encoding a Rab GTPase late endosome/multivesicular body marker, attenuates *P. infestans* infection and reduces the translocation of RXLR effector fusions from the pathogen to host cells.^60^ When considered together, these studies suggest that there may be a conserved mechanism involved in effector uptake by very diverse filamentous pathogens. There are, however, clearly distinct mechanisms of secretion in pathogens such as *Ustilago maydis*, in which a translocon has been implicated in secretion of at least a subset of its effectors,^61^ as well as in other fungal pathogens (for a review see Ref. [62]).

Once secreted, *M. oryzae* effectors take on distinct functions depending on their destination. The apoplastic LysM effector Slp1, for example, suppresses chitin‐triggered immunity by acting as a high affinity binder of chitin oligomers released by the pathogen which would normally elicit a pattern‐triggered immune response,^28^ requiring N‐glycosylation for its activity in the apoplast.^63^ Similarly, the chitinase Chia1 binds chitin in the apoplast to suppress immunity, but can also be recognised by the rice tetratricopeptide repeat protein OsTPR1 in a counter‐suppression strategy against the effector.^64^ An ascorbate oxidase, AO1, also affects apoplastic redox status and suppresses two immunity‐associated rice ascorbate oxidases.^65^ Cytoplasmic effectors meanwhile target distinct components of pattern‐triggered immunity signalling pathways. For example, mitochondria are targeted by at least two *M. oryzae* effectors. CDIP4 targets the mitochondria‐associated OsDjA9‐OsDRP1E protein complex to reduce rice immunity,^66^ while Avr‐Pita is a metalloprotease that has been reported to target the cytochrome *c* oxidase assembly protein OsCOX11, which regulates mitochondrial reactive oxygen species (ROS) metabolism.^67^ Effectors may also target processes such as plant exocytosis. The Avr‐Pii effector, for example, targets two rice Exo70s OsExo70F2 and OsExo70F3^68^ and defines a family of zinc‐finger effector fold (ZiF) effectors that bind to a specific Exo70 interface.^69^ Effectors can, however, also have multiple host targets. Avr‐Pii has, for instance, also been reported to interact with NADP‐malic enzyme (OsNADP‐ME2) to inhibit its activity,^70^ while the effector Avr‐Pizt is even more promiscuous and has been reported to suppress immunity by binding to the RING‐type ubiquitin E3 ligases APIP6 and APIP10,^71^, ^72^ the bZIP‐type transcription factor APIP5 to suppress APIP5‐triggered cell death,^73^ the nucleoporin protein APIP12^74^ and the potassium channel OsAKT1 by competing with protein kinase OsCIPK23 to modulate K^+^ channel activity.^75^ How Avr‐Pizt evolved to have such a large number of highly distinct interactors, however, is not clear. Effectors can also target large sets of immune‐related proteins such as heavy metal‐associated (HMA) domain, which fulfil diverse functions in immunity. The MAX effectors Avr1‐CO39, Avr‐PikD, and Pwl2, for example, all target HMA proteins and host plants have in turn evolved to recognise them during effector‐triggered immunity by integrating HMA domains into NLR immune receptors.^76^, ^77^, ^78^, ^79^, ^80^

## 7 FRONTIERS OF OUR UNDERSTANDING OF PATHOGENESIS BY *Magnaporthe oryzae*

A combination of molecular genetics, genomics, and live cell imaging has enabled rapid progress in our understanding of rice blast infection. We now have a basic knowledge of how an appressorium is formed, how it develops turgor and how the re‐polarisation process occurs to facilitate entry into the plant. However, there is much we still do not understand. The cell cycle control points that govern appressorium morphogenesis need precise definition and the genetic determinants need to be identified and characterised. These in turn need to be positioned in the context of the signalling pathways that we know regulate appressorium formation, the Pmk1 MAP kinase cascade in particular, but also the cAMP‐dependent protein kinase A, and the protein kinase C/cell integrity pathways, so that the network and associated checkpoints that govern appressorium development can be clearly defined. This will require live cell imaging to define the co‐localisation of individual components of these signalling pathways such that the spatial and temporal dynamics of the system can be understood. Definition of the Pmk1‐dependent phosphorylation landscape of appressorium development^15^ is a major advance that will enable the downstream targets of the pathway to be defined and connected with the transcriptional changes that have so far been identified (Osés‐Ruiz et al., 2021).^37^ However, a comprehensive analysis of Pmk1‐dependent transcriptional regulators is required to fully define the hierarchy of control that leads to infection cell morphogenesis. Appressorium turgor generation also requires deeper biochemical analysis as the synthetic pathway for compatible solute generation is still relatively poorly understood. Turgor control will also require the histidine phosphorylation landscape to be defined so that the role of Sln1^81^ can be fully understood. Appressorium re‐polarisation will also require the septin interactome to be described in detail so that polarity determinants and early acting virulence factors can be identified and the role of septins more clearly understood.^82^

During invasive growth the major challenge will be to determine the functions of more than 500 effector proteins. This seems a daunting task at this stage, given that we understand very few effector functions so far and those that have been studied, such as AvrPi‐zt, for example, can be complex involving multiple targets. Whether this is a common feature is currently unknown, but higher throughput gene functional analysis, perhaps using higher frequency CRISPR‐Cas9 genome editing will be required along with advances in live cell imaging at scale, to achieve this goal. Understanding effector uptake, BIC formation and the precise delivery mechanism is also an important goal for future research. We need to understand the consequences of effector function in eliciting major changes in host cell organisation and organelle distribution, for example, and in determining how such a large amount of additional plant membrane membrane is made in an infected cell to accommodate growing invasive hyphae. In this article, we have predominantly reported studies using the expression of functional fluorescent proteins visualised by laser scanning confocal microscopy to perform live cell imaging of effector and regulator localisation and to examine the rice‐*M. oryzae* interface.^23^, ^27^, ^28^, ^29^, ^30^, ^31^, ^83^, ^84^ Research questions regarding effector uptake and function, and host cellular responses during infection would, however, benefit greatly from advances in imaging platforms and microscopy techniques. Spinning disk confocal microscopy^85^, ^86^ with its high temporal resolution, for example, is ideal for imaging the dynamics of fast‐moving organelles such as mitochondria and endosomes during infection, which would greatly aid the analysis of effector delivery during infection. Multiphoton microscopy^83^, ^87^, ^88^, ^89^, ^90^, ^91^ which enables deep tissue imaging and minimises phototoxic damage, would particularly add value to the observation of structures located deeper in plant tissue such as the BIC to reveal its internal composition and the membrane dynamics of host‐pathogen interactions. Light sheet microscopy^92^, ^93^ offers the benefit of long‐term imaging due to low phototoxicity with a larger field of view. This could be applied, for instance, to monitoring hyphal development while looking at changes in host plant cell organisation, such as cytoskeletal re‐modelling, or in tracking fluorescent effector protein movement away from initial sites of infection. Live cell‐compatible super‐resolution microscopy platforms including structured illumination microscopy (SIM)^94^, ^95^, ^96^ and AiryScan microscopy^97^ would furthermore allow examination of host‐fungal interplay in greater detail, such as defining intricate details of the host endoplasmic reticulum during infection or localisation of proteins in plasmodesmata associated with immunity and the action of effectors in suppressing such responses (Fitzgibbon et al., 2010).^98^

Finally, these cell biology advances need to facilitate translation into new methods for disease control either via a more systematic effector‐guided deployment strategy for major resistance genes in durable combinations, based on an understanding of the prevailing pathogen population, or by means of better, targeted antifungal compounds that have limited environmental impact and can be made in a sustainable manner. These tasks are equally daunting, but exciting too, and can build on recent advances in our understanding of pathogenesis that have been driven by advances in live cell imaging of blast infections.

## Data Availability

Data sharing not applicable to this article as no data sets were generated or analysed during the current study.

## References

[1] Dean, R. , Van Kan, J. A. , Pretorius, Z. A. , Hammond‐Kosack, K. E. , Di Pietro, A. , Spanu, P. D. , Rudd, J. J. , Dickman, M. , Kahmann, R. , & Ellis, J. (2012). The top 10 fungal pathogens in molecular plant pathology. Molecular Plant Pathology, 13(4), 414–430. 22471698 10.1111/j.1364-3703.2011.00783.x PMC6638784

[2] Savary, S. , Willocquet, L. , Pethybridge, S. J. , Esker, P. , McRoberts, N. , & Nelson, A. (2019). The global burden of pathogens and pests on major food crops. Nature Ecology & Evolution, 3(3), 430–439. 10.1038/s41559-018-0793-y 30718852

[3] Nalley, L. , Tsiboe, F. , Durand‐Morat, A. , Shew, A. , & Thoma, G. (2016). Economic and Environmental Impact of Rice Blast Pathogen (*Magnaporthe oryzae*) Alleviation in the United States. PLoS ONE, 11(12), e0167295. 10.1371/journal.pone.0167295 27907101 PMC5131998

[4] Wilson, R. A. , & Talbot, N. J. (2009). Under pressure: Investigating the biology of plant infection by *Magnaporthe oryzae* . Nature Reviews Microbiology, 7(3), 185–195. 19219052 10.1038/nrmicro2032

[5] Khush, G. S. (2005). What it will take to feed 5.0 billion rice consumers in 2030. Plant Molecular Biology, 59, 1–6. 16217597 10.1007/s11103-005-2159-5

[6] Kato, H. , Yamamoto, M. , Yamaguchi‐Ozaki, T. , Kadouchi, H. , Iwamoto, Y. , Nakayashiki, H. , Tosa, Y. , Mayama, S. , & Mori, N. (2000). Pathogenicity, mating ability and DNA restriction fragment length polymorphisms of Pyricularia populations isolated from Gramineae, Bambusideae and Zingiberaceae plants. Journal of General Plant Pathology, 66, 30–47.

[7] Oh, H. , Tosa, Y. , Takabayashi, N. , Nakagawa, S. , Tomita, R. , Don, L. , Kusaba, M. , Nakayashiki, H. , & Mayama, S. (2002). Characterization of an Avena isolate of *Magnaporthe grisea* and identification of a locus conditioning its specificity on oat. Canadian Journal of Botany, 80(10), 1088–1095.

[8] Tosa, Y. , Hirata, K. , Tamba, H. , Nakagawa, S. , Chuma, I. , Isobe, C. , Osue, J. , Urashima, A. , Don, L. , & Kusaba, M. (2004). Genetic constitution and pathogenicity of Lolium isolates of *Magnaporthe oryzae* in comparison with host species‐specific pathotypes of the blast fungus. Phytopathology, 94(5), 454–462. 18943763 10.1094/PHYTO.2004.94.5.454

[9] Inoue, Y. , Vy, T. T. , Yoshida, K. , Asano, H. , Mitsuoka, C. , Asuke, S. , Anh, V. L. , Cumagun, C. J. , Chuma, I. , & Terauchi, R. (2017). Evolution of the wheat blast fungus through functional losses in a host specificity determinant. Science, 357(6346), 80–83. 28684523 10.1126/science.aam9654

[10] Islam, M. T. , Croll, D. , Gladieux, P. , Soanes, D. M. , Persoons, A. , Bhattacharjee, P. , Hossain, M. S. , Gupta, D. R. , Rahman, M. M. , & Mahboob, M. G. (2016). Emergence of wheat blast in Bangladesh was caused by a South American lineage of *Magnaporthe oryzae* . BMC biology, 14, 1–11. 27716181 10.1186/s12915-016-0309-7 PMC5047043

[11] Islam, M. T. , Kim, K.‐H. , & Choi, J. (2019). Wheat blast in Bangladesh: The current situation and future impacts. The Plant Pathology Journal, 35(1), 1. 30828274 10.5423/PPJ.RW.08.2018.0168 PMC6385656

[12] Latorre, S. M. , Were, V. M. , Foster, A. J. , Langner, T. , Malmgren, A. , Harant, A. , Asuke, S. , Reyes‐Avila, S. , Gupta, D. R. , Jensen, C. , Ma, W. , Mahmud, N. U. , Mehebub, M. S. , Mulenga, R. M. , Muzahid, A. N. M. , Paul, S. K. , Rabby, S. M. F. , Rahat, A. A. M. , Ryder, L. , … Kamoun, S. (2023). Genomic surveillance uncovers a pandemic clonal lineage of the wheat blast fungus. PLoS Biology, 21(4):e3002052. 10.1371/journal.pbio.3002052 37040332 PMC10089362

[13] Eseola, A. B. , Ryder, L. S. , Osés‐Ruiz, M. , Findlay, K. , Yan, X. , Cruz‐Mireles, N. , Molinari, C. , Garduño‐Rosales, M. , & Talbot, N. J. (2021). Investigating the cell and developmental biology of plant infection by the rice blast fungus *Magnaporthe oryzae* . Fungal Genetics and Biology, 154, 103562. 10.1016/j.fgb.2021.103562 33882359

[14] Ryder, L. S. , Lopez, S. G. , Michels, L. , Eseola, A. B. , Sprakel, J. , Ma, W. , & Talbot, N. J. (2023). A molecular mechanosensor for real‐time visualization of appressorium membrane tension in *Magnaporthe oryzae* . Nature Microbiology, 8(8), 1508–1519. 10.1038/s41564-023-01430-x PMC10390335 37474734

[15] Cruz‐Mireles, N. , Osés‐Ruiz, M. , Derbyshire, P. , Jégousse, C. , Ryder, L. S. , Bautista, M. J. A. , Eseola, A. , Sklenar, J. , Tang, B. , Yan, X. , Ma, W. , Findlay, K. C. , Were, V. , MacLean, D. , Talbot, N. J. , & Menke, F. L. H. (2024). The phosphorylation landscape of infection‐related development by the rice blast fungus. Cell, 187(10), 2557–2573.e2518. 10.1016/j.cell.2024.04.007 38729111

[16] Heath, M. C. , Valent, B. , Howard, R. J. , & Chumley, F. G. (1990). Interactions of two strains of *Magnaporthe grisea* with rice, goosegrass, and weeping lovegrass. Canadian Journal of Botany, 68(8), 1627–1637. 10.1139/b90-209

[17] Koga, H. , & Kobayashi, T. (1982). Comparison of the early infection process of *Pyricularia oryzae* Cav. in rice leaves of compatible and incompatible combinations. Japanese Journal of Phytopathology, 48(4), 506–513.

[18] Koga, H. , Kobayashi, T. , & Horino, O. (1982). Electron microscopical observation of rice leaves infected with *Pyricularia oryzae* Cav. in compatible and incompatible combinations I. Fine structure of invaded hyphae in host cells. Japanese Journal of Phytopathology, 48(3), 281–289.

[19] Peng, Y.‐L. , & Shishiyama, J. (1988). Temporal sequence of cytological events in rice leaves infected with *Pyricularia oryzae* . Canadian Journal of Botany, 66(4), 730–735.

[20] Peng, Y.‐L. , & Shishiyama, J. (1989). Timing of a cellular reaction in rice cultivars associated with differing degrees of resistance to Pyricularia oryzae. Canadian Journal of Botany, 67(9), 2704–2710.

[21] Koga, H. (1994). Hypersensitive death, autofluorescence, and ultrastructural changes in cells of leaf sheaths of susceptible and resistant near‐isogenic lines of rice (Pi‐zt) in relation to penetration and growth of Pyricularia oryzae. Canadian Journal of Botany, 72(10), 1463–1477.

[22] Sakamoto, M. (1949). On the new method of sheath‐inoculation of rice plants with blast fungus, *Pyricularia oryzae* Cav. for the study of the disease‐resistant nature of the plant. Bulletin of the Institute for Agricultural Research Tohoku University, Japan, 1, 120–129.

[23] Kankanala, P. , Czymmek, K. , & Valent, B. (2007). Roles for rice membrane dynamics and plasmodesmata during biotrophic invasion by the blast fungus. The Plant Cell, 19(2), 706–724. 10.1105/tpc.106.046300 17322409 PMC1867340

[24] Jones, K. , Kim, D. W. , Park, J. S. , & Khang, C. H. (2016). Live‐cell fluorescence imaging to investigate the dynamics of plant cell death during infection by the rice blast fungus *Magnaporthe oryzae* . BMC Plant Biology, 16(1), 69. 10.1186/s12870-016-0756-x 27000073 PMC4802709

[25] Shen, Q. , Liang, M. , Yang, F. , Deng, Y. Z. , & Naqvi, N. I. (2020). Ferroptosis contributes to developmental cell death in rice blast. New Phytologist, 227(6), 1831–1846. 32367535 10.1111/nph.16636

[26] Giraldo, M. C. , Dagdas, Y. F. , Gupta, Y. K. , Mentlak, T. A. , Yi, M. , Martinez‐Rocha, A. L. , Saitoh, H. , Terauchi, R. , Talbot, N. J. , & Valent, B. (2013). Two distinct secretion systems facilitate tissue invasion by the rice blast fungus *Magnaporthe oryzae* . Nature Communications, 4, 1996. 10.1038/ncomms2996 PMC3709508 23774898

[27] Khang, C. H. , Berruyer, R. , Giraldo, M. C. , Kankanala, P. , Park, S. Y. , Czymmek, K. , Kang, S. , & Valent, B. (2010). Translocation of *Magnaporthe oryzae* effectors into rice cells and their subsequent cell‐to‐cell movement. Plant Cell, 22(4), 1388–1403. 10.1105/tpc.109.069666 20435900 PMC2879738

[28] Mentlak, T. A. , Kombrink, A. , Shinya, T. , Ryder, L. S. , Otomo, I. , Saitoh, H. , Terauchi, R. , Nishizawa, Y. , Shibuya, N. , Thomma, B. P. H. J. , & Talbot, N. J. (2012). Effector‐mediated suppression of chitin‐triggered immunity by *Magnaporthe oryzae* Is necessary for rice blast disease. The Plant Cell, 24(1), 322–335. 10.1105/tpc.111.092957 22267486 PMC3289562

[29] Mochizuki, S. , Minami, E. , & Nishizawa, Y. (2015). Live‐cell imaging of rice cytological changes reveals the importance of host vacuole maintenance for biotrophic invasion by blast fungus, *Magnaporthe oryzae* . MicrobiologyOpen, 4(6), 952–966. 10.1002/mbo3.304 26472068 PMC4694143

[30] Oliveira‐Garcia, E. , Tamang, T. M. , Park, J. , Dalby, M. , Martin‐Urdiroz, M. , Rodriguez Herrero, C. , Vu, A. H. , Park, S. , Talbot, N. J. , & Valent, B. (2023). Clathrin‐mediated endocytosis facilitates the internalization of *Magnaporthe oryzae* effectors into rice cells. The Plant Cell, 35(7), 2527–2551. 10.1093/plcell/koad094 36976907 PMC10291035

[31] Yan, X. , Tang, B. , Ryder, L. S. , MacLean, D. , Were, V. M. , Eseola, A. B. , Cruz‐Mireles, N. , Ma, W. , Foster, A. J. , Osés‐Ruiz, M. , & Talbot, N. J. (2023). The transcriptional landscape of plant infection by the rice blast fungus *Magnaporthe oryzae* reveals distinct families of temporally co‐regulated and structurally conserved effectors. The Plant Cell, 35(5), 1360–1385. 10.1093/plcell/koad036 36808541 PMC10118281

[32] Hamer, J. E. , Howard, R. J. , Chumley, F. G. , & Valent, B. (1988). A mechanism for surface attachment in spores of a plant pathogenic fungus. Science, 239(4837), 288–290. 10.1126/science.239.4837.288 17769992

[33] Fernandez, J. , & Orth, K. (2018). Rise of a cereal killer: The biology of *Magnaporthe oryzae* biotrophic growth. Trends in Microbiology, 26(7), 582–597. 10.1016/j.tim.2017.12.007 29395728 PMC6003838

[34] Howard, R. J. , & Ferrari, M. A. (1989). Role of melanin in appressorium function. Experimental Mycology, 13(4), 403–418.

[35] Howard, R. J. , & Valent, B. (1996). Breaking and entering: host penetration by the fungal rice blast pathogen *Magnaporthe grisea* . Annual Review of Microbiology, 50(1996), 491–512. 10.1146/annurev.micro.50.1.491 8905089

[36] Talbot, N. J. (2003). On the trail of a cereal killer: Exploring the biology of *Magnaporthe grisea* . Annual Review of Microbiology, 57, 177–202. 10.1146/annurev.micro.57.030502.090957 14527276

[37] Osés‐Ruiz, M. , Cruz‐Mireles, N. , Martin‐Urdiroz, M. , Soanes, D. M. , Eseola, A. B. , Tang, B. , Derbyshire, P. , Nielsen, M. , Cheema, J. , Were, V. , Eisermann, I. , Kershaw, M. J. , Yan, X. , Valdovinos‐Ponce, G. , Molinari, C. , Littlejohn, G. R. , Valent, B. , Menke, F. L. H. , & Talbot, N. J. (2021). Appressorium‐mediated plant infection by *Magnaporthe oryzae* is regulated by a Pmk1‐dependent hierarchical transcriptional network. Nature Microbiology, 6(11), 1383–1397. 10.1038/s41564-021-00978-w 34707224

[38] de Jong, J. C. , McCormack, B. J. , Smirnoff, N. , & Talbot, N. J. (1997). Glycerol generates turgor in rice blast. Nature, 389(6648), 244–244. 10.1038/38418

[39] Howard, R. J. , Ferrari, M. A. , Roach, D. H. , & Money, N. P. (1991). Penetration of hard substrates by a fungus employing enormous turgor pressures. PNAS, 88(24), 11281–11284. 10.1073/pnas.88.24.11281 1837147 PMC53118

[40] Saunders, D. G. , Dagdas, Y. F. , & Talbot, N. J. (2010). Spatial uncoupling of mitosis and cytokinesis during appressorium‐mediated plant infection by the rice blast fungus *Magnaporthe oryzae* . Plant Cell, 22(7), 2417–2428. 10.1105/tpc.110.074492 20639448 PMC2929119

[41] Marroquin‐Guzman, M. , & Wilson, R. A. (2015). GATA‐dependent glutaminolysis drives appressorium formation in *Magnaporthe oryzae* by suppressing TOR inhibition of cAMP/PKA signaling. PLOS Pathogens, 11(4), e1004851. 10.1371/journal.ppat.1004851 25901357 PMC4406744

[42] Kershaw, M. J. , & Talbot, N. J. (2009). Genome‐wide functional analysis reveals that infection‐associated fungal autophagy is necessary for rice blast disease. Proceedings of the National Academy of Sciences, 106(37), 15967–15972. 10.1073/pnas.0901477106 PMC2747227 19717456

[43] Veneault‐Fourrey, C. , Barooah, M. , Egan, M. , Wakley, G. , & Talbot, N. J. (2006). Autophagic fungal cell death is necessary for infection by the rice blast fungus. Science, 312(5773), 580–583. 10.1126/science.1124550 16645096

[44] Li, G. , Dulal, N. , Gong, Z. , & Wilson, R. A. (2023). Unconventional secretion of *Magnaporthe oryzae* effectors in rice cells is regulated by tRNA modification and codon usage control. Nature microbiology, 8(9), 1706–1716. 10.1038/s41564-023-01443-6 37563288

[45] Liu, Q. , Long, R. , Lin, C. , Bi, X. , Liang, Z. , & Deng, Y. Z. (2024). Phosphatidylethanolamines link ferroptosis and autophagy during appressorium formation of rice blast fungus. Molecular Plant Pathology, 25(7), e13489. 10.1111/mpp.13489 38956897 PMC11219472

[46] Dagdas, Y. F. , Yoshino, K. , Dagdas, G. , Ryder, L. S. , Bielska, E. , Steinberg, G. , & Talbot, N. J. (2012). Septin‐mediated plant cell invasion by the rice blast fungus, *Magnaporthe oryzae* . Science, 336(6088), 1590–1595. 22723425 10.1126/science.1222934

[47] Dulal, N. , Rogers, A. M. , Proko, R. , Bieger, B. D. , Liyanage, R. , Krishnamurthi, V. R. , Wang, Y. , & Egan, M. J. (2021). Turgor‐dependent and coronin‐mediated F‐actin dynamics drive septin disc‐to‐ring remodeling in the blast fungus *Magnaporthe oryzae* . Journal of Cell Science, 134(5), jcs251298. 33414165 10.1242/jcs.251298

[48] Gupta, Y. K. , Dagdas, Y. F. , Martinez‐Rocha, A.‐L. , Kershaw, M. J. , Littlejohn, G. R. , Ryder, L. S. , Sklenar, J. , Menke, F. , & Talbot, N. J. (2015). Septin‐dependent assembly of the exocyst is essential for plant infection by *Magnaporthe oryzae* . The Plant Cell, 27(11), 3277–3289. 26566920 10.1105/tpc.15.00552 PMC4682301

[49] Sakulkoo, W. , Osés‐Ruiz, M. , Oliveira Garcia, E. , Soanes, D. M. , Littlejohn, G. R. , Hacker, C. , Correia, A. , Valent, B. , & Talbot, N. J. (2018). A single fungal MAP kinase controls plant cell‐to‐cell invasion by the rice blast fungus. Science, 359(6382), 1399–1403. 10.1126/science.aaq0892 29567712

[50] Cruz‐Mireles, N. , Eseola, A. B. , Osés‐Ruiz, M. , Ryder, L. S. , & Talbot, N. J. (2021). From appressorium to transpressorium – Defining the morphogenetic basis of host cell invasion by the rice blast fungus. PLOS Pathogens, 17(7), e1009779. 10.1371/journal.ppat.1009779 34329369 PMC8323886

[51] Ryder, L. S. , Cruz‐Mireles, N. , Molinari, C. , Eisermann, I. , Eseola, A. B. , & Talbot, N. J. (2022). The appressorium at a glance. Journal of Cell Science, 135(14), jcs259857. 10.1242/jcs.259857 35856284

[52] Koga, H. , Dohi, K. , Nakayachi, O. , & Mori, M. (2004). A novel inoculation method of *Magnaporthe grisea* for cytological observation of the infection process using intact leaf sheaths of rice plants. Physiological and Molecular Plant Pathology, 64(2), 67–72. 10.1016/j.pmpp.2004.07.002

[53] de Jonge, R. , Bolton, M. D. , & Thomma, B. P. (2011). How filamentous pathogens co‐opt plants: The ins and outs of fungal effectors. Current Opinion in Plant Biology, 14(4), 400–406. 10.1016/j.pbi.2011.03.005 21454120

[54] Yang, T. , Song, L. , Hu, J. , Qiao, L. , Yu, Q. , Wang, Z. , Chen, X. , & Lu, G.‐d. (2024). *Magnaporthe oryzae* effector AvrPik‐D targets a transcription factor WG7 to suppress rice immunity. Rice, 17(1), 14. 10.1186/s12284-024-00693-0 38351214 PMC10864242

[55] Zhang, S. , & Xu, J.‐R. (2014). Effectors and effector delivery in *Magnaporthe oryzae* . PLOS Pathogens, 10(1), e1003826. 10.1371/journal.ppat.1003826 24391496 PMC3879361

[56] Tang, B. , Yan, X. , Ryder, L. S. , Bautista, M. J. A. , Cruz‐Mireles, N. , Soanes, D. M. , Molinari, C. , Foster, A. J. , & Talbot, N. J. (2023). Rgs1 is a regulator of effector gene expression during plant infection by the rice blast fungus *Magnaporthe oryzae* . Proceedings of the National Academy of Sciences, 120(12), e2301358120. 10.1073/pnas.2301358120 PMC10041150 36913579

[57] Mosquera, G. , Giraldo, M. C. , Khang, C. H. , Coughlan, S. , & Valent, B. (2009). Interaction transcriptome analysis identifies *Magnaporthe oryzae* BAS1‐4 as Biotrophy‐associated secreted proteins in rice blast disease. Plant Cell, 21(4), 1273–1290. 10.1105/tpc.107.055228 19357089 PMC2685627

[58] Li, G. , Gong, Z. , Dulal, N. , Marroquin‐Guzman, M. , Rocha, R. O. , Richter, M. , & Wilson, R. A. (2023). A protein kinase coordinates cycles of autophagy and glutaminolysis in invasive hyphae of the fungus *Magnaporthe oryzae* within rice cells. Nature Communications, 14(1), 4146. 10.1038/s41467-023-39880-w PMC10338429 37438395

[59] Wang, S. , Boevink, P. C. , Welsh, L. , Zhang, R. , Whisson, S. C. , & Birch, P. R. J. (2017). Delivery of cytoplasmic and apoplastic effectors from Phytophthora infestans haustoria by distinct secretion pathways. New Phytologist, 216(1), 205–215. 10.1111/nph.14696 28758684 PMC5601276

[60] Wang, H. , Wang, S. , Wang, W. , Xu, L. , Welsh, L. R. J. , Gierlinski, M. , Whisson, S. C. , Hemsley, P. A. , Boevink, P. C. , & Birch, P. R. J. (2023). Uptake of oomycete RXLR effectors into host cells by clathrin‐mediated endocytosis. The Plant Cell, 35(7), 2504–2526. 10.1093/plcell/koad069 36911990 PMC10291037

[61] Ludwig, N. , Reissmann, S. , Schipper, K. , Gonzalez, C. , Assmann, D. , Glatter, T. , Moretti, M. , Ma, L.‐S. , Rexer, K.‐H. , Snetselaar, K. , & Kahmann, R. (2021). A cell surface‐exposed protein complex with an essential virulence function in *Ustilago maydis* . Nature Microbiology, 6(6), 722–730. 10.1038/s41564-021-00896-x PMC8159752 33941900

[62] Lo Presti, L. , & Kahmann, R. (2017). How filamentous plant pathogen effectors are translocated to host cells. Current Opinion in Plant Biology, 38, 19–24. 10.1016/j.pbi.2017.04.005 28460240

[63] Chen, X. L. , Shi, T. , Yang, J. , Shi, W. , Gao, X. , Chen, D. , Xu, X. , Xu, J. R. , Talbot, N. J. , & Peng, Y. L. (2014). N‐glycosylation of effector proteins by an α‐1,3‐mannosyltransferase is required for the rice blast fungus to evade host innate immunity. Plant Cell, 26(3), 1360–1376. 10.1105/tpc.114.123588 24642938 PMC4001389

[64] Yang, C. , Yu, Y. , Huang, J. , Meng, F. , Pang, J. , Zhao, Q. , Islam, M. A. , Xu, N. , Tian, Y. , & Liu, J. (2019). Binding of the *Magnaporthe oryzae* Chitinase MoChia1 by a rice tetratricopeptide repeat protein allows free chitin to trigger immune responses. The Plant Cell, 31(1), 172–188. 10.1105/tpc.18.00382 30610168 PMC6391695

[65] Hu, J. , Liu, M. , Zhang, A. , Dai, Y. , Chen, W. , Chen, F. , Wang, W. , Shen, D. , Telebanco‐Yanoria, M. J. , Ren, B. , Zhang, H. , Zhou, H. , Zhou, B. , Wang, P. , & Zhang, Z. (2022). Co‐evolved plant and blast fungus ascorbate oxidases orchestrate the redox state of host apoplast to modulate rice immunity. Molecular Plant, 15(8), 1347–1366. 10.1016/j.molp.2022.07.001 35799449 PMC11163382

[66] Xu, G. , Zhong, X. , Shi, Y. , Liu, Z. , Jiang, N. , Liu, J. , Ding, B. , Li, Z. , Kang, H. , Ning, Y. , Liu, W. , Guo, Z. , Wang, G.‐L. , & Wang, X. (2020). A fungal effector targets a heat shock–dynamin protein complex to modulate mitochondrial dynamics and reduce plant immunity. Science Advances, 6(48), eabb7719. 10.1126/sciadv.abb7719 33239288 PMC7688324

[67] Han, J. , Wang, X. , Wang, F. , Zhao, Z. , Li, G. , Zhu, X. , Su, J. , & Chen, L. (2021). The fungal effector Avr‐Pita suppresses innate immunity by increasing COX activity in rice mitochondria. Rice, 14(1), 12. 10.1186/s12284-021-00453-4 33443630 PMC7809080

[68] Fujisaki, K. , Abe, Y. , Ito, A. , Saitoh, H. , Yoshida, K. , Kanzaki, H. , Kanzaki, E. , Utsushi, H. , Yamashita, T. , Kamoun, S. , & Terauchi, R. (2015). Rice Exo70 interacts with a fungal effector, AVR‐Pii, and is required for AVR‐Pii‐triggered immunity. The Plant Journal, 83(5), 875–887. 10.1111/tpj.12934 26186703

[69] De la Concepcion, J. C. , Fujisaki, K. , Bentham, A. R. , Cruz Mireles, N. , Sanchez de Medina Hernandez, V. , Shimizu, M. , Lawson, D. M. , Kamoun, S. , Terauchi, R. , & Banfield, M. J. (2022). A blast fungus zinc‐finger fold effector binds to a hydrophobic pocket in host Exo70 proteins to modulate immune recognition in rice. PNAS, 119(43), e2210559119. 10.1073/pnas.2210559119 36252011 PMC9618136

[70] Singh, R. , Dangol, S. , Chen, Y. , Choi, J. , Cho, Y.‐S. , Lee, J.‐E. , Choi, M.‐O. , & Jwa, N.‐S. (2016). *Magnaporthe oryzae* effector AVR‐Pii helps to establish compatibility by inhibition of the rice NADP‐malic enzyme resulting in disruption of oxidative burst and host innate immunity. Molecules and Cells, 39(5), 426–438. 10.14348/molcells.2016.0094 27126515 PMC4870191

[71] Park, C.‐H. , Chen, S. , Shirsekar, G. , Zhou, B. , Khang, C. H. , Songkumarn, P. , Afzal, A. J. , Ning, Y. , Wang, R. , Bellizzi, M. , Valent, B. , & Wang, G.‐L. (2012). The *Magnaporthe oryzae* effector AvrPiz‐t targets the RING E3 ubiquitin ligase APIP6 to suppress pathogen‐associated molecular pattern–triggered immunity in rice. The Plant Cell, 24(11), 4748–4762. 10.1105/tpc.112.105429 23204406 PMC3531864

[72] Park, C. H. , Shirsekar, G. , Bellizzi, M. , Chen, S. , Songkumarn, P. , Xie, X. , Shi, X. , Ning, Y. , Zhou, B. , Suttiviriya, P. , Wang, M. , Umemura, K. , & Wang, G.‐L. (2016). The E3 ligase APIP10 connects the effector AvrPiz‐t to the NLR receptor Piz‐t in rice. PLOS Pathogens, 12(3), e1005529. 10.1371/journal.ppat.1005529 27031246 PMC4816579

[73] Wang, R. , Ning, Y. , Shi, X. , He, F. , Zhang, C. , Fan, J. , Jiang, N. , Zhang, Y. , Zhang, T. , Hu, Y. , Bellizzi, M. , & Wang, G.‐L. (2016). Immunity to rice blast disease by suppression of effector‐triggered necrosis. Current Biology, 26(18), 2399–2411. 10.1016/j.cub.2016.06.072 27641772

[74] Tang, M. , Ning, Y. , Shu, X. , Dong, B. , Zhang, H. , Wu, D. , Wang, H. , Wang, G.‐L. , & Zhou, B. (2017). The Nup98 homolog APIP12 targeted by the effector AvrPiz‐t is involved in rice basal resistance against *Magnaporthe oryzae* . Rice, 10(1), 5. 10.1186/s12284-017-0144-7 28205154 PMC5311014

[75] Shi, X. , Long, Y. , He, F. , Zhang, C. , Wang, R. , Zhang, T. , Wu, W. , Hao, Z. , Wang, Y. , Wang, G.‐L. , & Ning, Y. (2018). The fungal pathogen *Magnaporthe oryzae* suppresses innate immunity by modulating a host potassium channel. PLOS Pathogens, 14(1), e1006878. 10.1371/journal.ppat.1006878 29385213 PMC5809103

[76] Guo, L. , Cesari, S. , de Guillen, K. , Chalvon, V. , Mammri, L. , Ma, M. , Meusnier, I. , Bonnot, F. , Padilla, A. , Peng, Y. L. , Liu, J. , & Kroj, T. (2018). Specific recognition of two MAX effectors by integrated HMA domains in plant immune receptors involves distinct binding surfaces. PNAS, 115(45), 11637–11642. 10.1073/pnas.1810705115 30355769 PMC6233088

[77] Maidment, J. H. R. , Franceschetti, M. , Maqbool, A. , Saitoh, H. , Jantasuriyarat, C. , Kamoun, S. , Terauchi, R. , & Banfield, M. J. (2021). Multiple variants of the fungal effector AVR‐Pik bind the HMA domain of the rice protein OsHIPP19, providing a foundation to engineer plant defense. Journal of Biological Chemistry, 296, 100371. 10.1016/j.jbc.2021.100371 33548226 PMC7961100

[78] Maqbool, A. , Saitoh, H. , Franceschetti, M. , Stevenson, C. E. M. , Uemura, A. , Kanzaki, H. , Kamoun, S. , Terauchi, R. , & Banfield, M. J. (2015). Structural basis of pathogen recognition by an integrated HMA domain in a plant NLR immune receptor. Elife, 4, e08709. 10.7554/eLife.08709 26304198 PMC4547098

[79] Were, V. , Yan, X. , Foster, A. J. , Sklenar, J. , Langner, T. , Bentham, A. , Zdrzałek, R. , Ryder, L. , Kaimenyi, D. , Gomez De La Cruz, D. , Gentle, A. , Petit‐Houdenot, Y. , Eseola, A. B. , Smoker, M. , Bautista, M. J. , Ma, W. , Kourelis, J. , Maclean, D. , Banfield, M. J. , … Talbot, N. J. (2024). The blast effector Pwl2 is a virulence factor that modifies the cellular localisation of host protein HIPP43 to suppress immunity. BioRxiv, 2024.2001.2020.576406. 10.1101/2024.01.20.576406

[80] Zdrzałek, R. , Xi, Y. , Langner, T. , Bentham, A. R. , Petit‐Houdenot, Y. , De la Concepcion, J. C. , Harant, A. , Shimizu, M. , Were, V. , Talbot, N. J. , Terauchi, R. , Kamoun, S. , & Banfield, M. J. (2024). Bioengineering a plant NLR immune receptor with a robust binding interface toward a conserved fungal pathogen effector. Proceedings of the National Academy of Sciences, 121(28), e2402872121. 10.1073/pnas.2402872121 PMC11252911 38968126

[81] Ryder, L. S. , Dagdas, Y. F. , Kershaw, M. J. , Venkataraman, C. , Madzvamuse, A. , Yan, X. , Cruz‐Mireles, N. , Soanes, D. M. , Oses‐Ruiz, M. , Styles, V. , Sklenar, J. , Menke, F. L. H. , & Talbot, N. J. (2019). A sensor kinase controls turgor‐driven plant infection by the rice blast fungus. Nature, 574(7778), 423–427. 10.1038/s41586-019-1637-x 31597961

[82] Foster, A. J. , Martin‐Urdiroz, M. , Yan, X. , Wright, H. S. , Soanes, D. M. , & Talbot, N. J. (2018). CRISPR‐Cas9 ribonucleoprotein‐mediated co‐editing and counterselection in the rice blast fungus. Scientific Reports, 8(1), 14355. 10.1038/s41598-018-32702-w 30254203 PMC6156577

[83] Czymmek, K. J. , Bourett, T. M. , Sweigard, J. A. , Carroll, A. , & Howard, R. J. (2002). Utility of cytoplasmic fluorescent proteins for live‐cell imaging of *Magnaporthe grisea* in planta. Mycologia, 94(2), 280–289. 10.1080/15572536.2003.11833234 21156498

[84] Giraldo, M. C. , & Valent, B. (2013). Filamentous plant pathogen effectors in action. Nature Reviews Microbiology, 11(11), 800–814. 10.1038/nrmicro3119 24129511

[85] Henty‐Ridilla, J. L. , Shimono, M. , Li, J. , Chang, J. H. , Day, B. , & Staiger, C. J. (2013). The plant actin cytoskeleton responds to signals from microbe‐associated molecular patterns. Plos Pathogens, 9(4), e1003290. 10.1371/journal.ppat.1003290 23593000 PMC3616984

[86] Oreopoulos, J. , Berman, R. , & Browne, M. (2014). Spinning‐disk confocal microscopy: Present technology and future trends. Methods in Cell Biology, 123, 153–175. 10.1016/b978-0-12-420138-5.00009-4 24974027

[87] Bourett, T. M. , Sweigard, J. A. , Czymmek, K. J. , Carroll, A. , & Howard, R. J. (2002). Reef coral fluorescent proteins for visualizing fungal pathogens. Fungal Genetics and Biology, 37(3), 211–220. 10.1016/s1087-1845(02)00524-8 12431456

[88] Czymmek, K. J. , Fogg, M. , Powell, D. H. , Sweigard, J. , Park, S.‐Y. , & Kang, S. (2007). In vivo time‐lapse documentation using confocal and multi‐photon microscopy reveals the mechanisms of invasion into the Arabidopsis root vascular system by *Fusarium oxysporum* . Fungal Genetics and Biology, 44(10), 1011–1023. 10.1016/j.fgb.2007.01.012 17379550

[89] Larson, A. M. (2011). Multiphoton microscopy. Nature Photonics, 5(1), 1–1. 10.1038/nphoton.an.2010.2

[90] Mizuta, Y. (2021). Advances in two‐photon imaging in plants. Plant and Cell Physiology, 62(8), 1224–1230. 10.1093/pcp/pcab062 34019083 PMC8579158

[91] Mizuta, Y. , Kurihara, D. , & Higashiyama, T. (2015). Two‐photon imaging with longer wavelength excitation in intact Arabidopsis tissues. Protoplasma, 252(5), 1231–1240. 10.1007/s00709-014-0754-5 25588923

[92] Keller, P. J. , & Dodt, H. U. (2012). Light sheet microscopy of living or cleared specimens. Current Opinion in Neurobiology, 22(1), 138–143. 10.1016/j.conb.2011.08.003 21925871

[93] Ovečka, M. , Sojka, J. , Tichá, M. , Komis, G. , Basheer, J. , Marchetti, C. , Šamajová, O. , Kuběnová, L. , & Šamaj, J. (2022). Imaging plant cells and organs with light‐sheet and super‐resolution microscopy. Plant Physiology, 188(2), 683–702. 10.1093/plphys/kiab349 35235660 PMC8825356

[94] Gustafsson, M. G. (2000). Surpassing the lateral resolution limit by a factor of two using structured illumination microscopy. Journal of Microscopy, 198(Pt 2), 82–87. 10.1046/j.1365-2818.2000.00710.x 10810003

[95] Lin, R. , Kipreos, E. T. , Zhu, J. , Khang, C. H. , & Kner, P. (2021). Subcellular three‐dimensional imaging deep through multicellular thick samples by structured illumination microscopy and adaptive optics. Nature Communications, 12(1), 3148. 10.1038/s41467-021-23449-6 PMC8149693 34035309

[96] Wu, Y. , & Shroff, H. (2018). Faster, sharper, and deeper: Structured illumination microscopy for biological imaging. Nature Methods, 15(12), 1011–1019. 10.1038/s41592-018-0211-z 30478322

[97] Huff, J. (2015). The Airyscan detector from ZEISS: Confocal imaging with improved signal‐to‐noise ratio and super‐resolution. Nature Methods, 12(12), i–ii. 10.1038/nmeth.f.388

[98] Fitzgibbon, J. , Bell, K. , King, E. , & Oparka, K. (2010). Super‐resolution imaging of plasmodesmata using three‐dimensional structured illumination microscopy. Plant Physiology, 153(4), 1453–1463. 10.1104/pp.110.157941 20508140 PMC2923914

